# Dyslipidemia Assessed in Pediatric Patients: Validation of LDL-C Assessed by Friedewald Formula, Direct Assessment, and Sampson–NIH Formula

**DOI:** 10.3390/diagnostics15232979

**Published:** 2025-11-24

**Authors:** Joanna Wawer, Agnieszka Chojęta, Jakub Swadźba, Mariola Janiszewska, Michał Chojęta, Genowefa Anna Wawer, Ewelina Grywalska, Anna Milaniuk

**Affiliations:** 1 Laboratory of Experimental Immunology, Medical University of Lublin, 20-059 Lublin, Poland; ewelina.grywalska@umlub.edu.pl; 2 Hospital Laboratory, University Pediatric Hospital, 20-093 Lublin, Poland; agnieszka.chojeta@umlub.edu.pl; 3 Endocrinology and Metabolic Laboratory, Department of Pediatric Endocrinology and Diabetology, Medical University of Lublin, 20-059 Lublin, Poland; 4 Medical Faculty, Andrzej Frycz Modrzewski Kraków University, 30-705 Kraków, Poland; jakub.swadzba@diag.pl; 5 Medical Department Diagnostyka S.A., 31-864 Kraków, Poland; 6 Department of Medical Informatics and Statistics with the e-Health Laboratory, Faculty of Medical Sciences, Medical University of Lublin, 20-059 Lublin, Poland; mariola.janiszewska@umlub.edu.pl; 7 MedAI Student Scientific Club, Department of Medical Informatics and Statistics with the e-Health, Medical University of Lublin, 20-081 Lublin, Poland; michalchojeta9@gmail.com; 8 Department of Foreign Languages, Medical University of Lublin, 20-059 Lublin, Poland; genowefawawer@umlub.edu.pl; 9 Department of Hematology, Oncology and Transplantology, Medical University of Lublin, 20-059 Lublin, Poland; anna_milaniuk@wp.pl

**Keywords:** LDL-C, Friedewald formula, direct assessment, Sampson–NIH formula, cardiac risk

## Abstract

**Background**: The epidemic increase in obesity, metabolic syndrome, cardiac disease, or hypertension is associated with lipid deregulation. Studies suggest a strong link between elevated levels of plasma cholesterol and the premature formation of atherosclerotic plaques. Primary prevention of early clinical manifestations of atherosclerosis allows slowing or preventing the development of several health problems later in adult life. **Objectives**: The purpose of this study was the validation of LDL-C measured by the Friedewald formula, direct method, and Sampson–NIH Formula. The results of the three methods used to assess LDL-C were compared to check whether the three measurements of LDL-C yielded different results. **Methods**: The study was conducted in a large cohort of in-patients aged 8 months to 18 years. Lipid profile parameters were determined. Indirect methods for dyslipidemia diagnosis were compared against direct LDL measurement. Incorrect and missed diagnoses were analyzed. To measure the central tendency, a statistical analysis of distributions of numerical variables was used. Differences between categorical variables were assessed. The agreement between pairs of competing methods in estimating LDL concentration was assessed via Bland–Altman analysis. **Results**: In total, 1982 pediatric patients underwent lipid profile assessment. Significant differences in lipid parameters between boys and girls were observed. TG, TC, and HDL levels were higher in boys. LDL-C as measured by the Friedewald formula and direct methods showed significant differences. Comparison of the direct methods with the Sampson–NIH indicated that the Sampson–NIH formula underestimates LDL values. **Conclusions**: The analysis revealed differences between the methods used to assess dyslipidemia. A systematic underestimation of LDL concentrations determined by the indirect methods was found. Small differences between the Friedewald and Sampson–NIH methods were observed. Although both indirect methods underestimate LDL levels compared to the direct method, the differences between them are small, though still detectable.

## 1. Introduction

### 1.1. Dyslipidemia

Dyslipidemia is defined as abnormalities in one or more of the lipid profile parameters, i.e., total cholesterol (TC), low-density lipoprotein (LDL-C), non-high-density lipoprotein (non-HDL-C), triglycerides (TG), and high-density lipoprotein (HDL-C). Considering the fundamental role of lipids in many body functions, any imbalance can produce a range of diseases, e.g., high levels of LDL-C (the “bad” cholesterol) can lead to atherosclerotic plaque build-up in the arteries, increasing the risk of cardiovascular diseases. The HDL fraction is believed to have multidirectional anti-atherosclerotic effects: anti-inflammatory, antithrombotic, antioxidant, antiapoptotic, and profibrinolytic [1,2]. Studies suggest a strong link between elevated levels of plasma cholesterol and premature formation of atherosclerotic plaques. The health condition of a newborn results from the course of intrauterine development and is decisive for the child’s further development and quality of life in adult life [3]. Thus, identification of dyslipidemia in pediatric patients is essential for early diagnosis and treatment in childhood as well as to prevent its progression in adulthood [4].

### 1.2. Primary and Secondary Dyslipidemias

According to the European Atherosclerosis Society, three types of dyslipidemia are distinguished, i.e., hypercholesterolemia (increased levels of TC and LDL-C), hypertriglyceridemia (increased levels of TG and VLDL-C), and mixed hyperlipidemia (increased levels of TC and TG). Each of these disorders may have primary or secondary etiology. Primary hyperlipidemias are genetically determined. This group includes, e.g., homo- and heterozygous familial hypercholesterolemia, familial apolipoprotein B-100 defect, familial lipoprotein lipase deficiency, familial mixed hyperlipidemia, chylomicronemia syndrome, low HDL-C syndrome, and other rare disorders. Secondary hyperlipidemias result from metabolic disorders caused by other diseases, drugs, or environmental factors [5].

The etiology of dyslipidemia is influenced by diet, lifestyle, level of physical activity, concomitant medical conditions, and/or genetic predisposition. Pediatric patients with a higher BMI (85–95th percentiles) are more likely to suffer from dyslipidemia [6]. The epidemic of obesity, metabolic syndrome, cardiovascular disease, or hypertension is associated with lipid abnormalities. Researchers suggest that the evidence of atherosclerotic lesions developed in childhood is directly associated with aggressive treatment of lipid disorders in adults, which emphasizes the need for early treatment [7].

Primary prevention of early clinical manifestations of atherosclerosis allows slowing or preventing the premature development of several health problems later in adult life. Hence, the strategy of targeted screening for dyslipidemia in children has been postulated. Numerous factors, including metabolic, genetic, and environmental conditions along with age, sex, and ethnicity, are likely to affect plasma lipids and lipoproteins concentrations in children [8]. Moreover, several risk factors such as obesity, hypertension, diabetes, smoking, poor dietary habits, and sedentary lifestyle can promote atherosclerosis later in adult life. Scientists and clinicians postulate either targeted or selective screening of children for dyslipidemia [9].

The best strategy, however, seems to be a universal screening in all or as many children as possible. Insufficient knowledge of the value of the lipid profile and health risks and overall health condition among parents speak to the importance of that approach [10]. Early screening can help select children with undiagnosed hereditary dyslipidemia and uncover their cardiovascular risk [8]. According to the National Heart, Lung, and Blood Institute (NHLBI) guidelines, targeted or selective screening should be directed toward children as young as aged 2–10 years with a positive family or personal history. Universal screening is recommended for all children aged 9 to 11 years. It is not recommended during puberty or in early adolescence (12–16 year olds), as it can produce falsely low results due to reduced lipid production during this time. Universal screening is also recommended for 17–21-year-olds [9]. Polish recommendations for the management of familial hypercholesterolemia (FH) state that early diagnosis and prompt treatment of FH in children can reduce the risk of cardiovascular problems in adults. That justifies the screening of the pediatric population for lipid disorders. Moreover, such a strategy could significantly reduce the cost of treating complications associated with untreated hypercholesterolemia [11].

### 1.3. Lipid Profile

In medical diagnostics, a lipid profile consists of the determination/calculation of serum/plasma concentration TC, HDL-C, LDL-C, TG, and non-HDL-C. Among these, the most important is LDL-C concentration [12]. Understanding a patient’s lipid profile provides a deep insight into their cardiovascular well-being and eventually leads to conscious, life-altering decisions. Family doctors suggest patients should have routine health check-ups to detect potential risk as early as possible, especially in individuals diagnosed with diabetes, hypertension, or other conditions that increase the risk of heart disease or stroke [13].

The guidelines of the Polish Society of Laboratory Diagnostics and the Polish Lipid Association on laboratory diagnostics of lipid metabolism disorders encourage laboratory diagnosticians and physicians managing patients with lipid disorders to follow the guidelines when making a clinical assessment when they define and implement medical prophylactic strategies, diagnosis, or treatment. An assessment of lipid profile should be made in conditions of everyday activity, health, and nutrition of the examined individual. Blood samples for routine lipid profile testing do not have to be taken on an empty stomach. Retesting in a fasting state should be considered at TG concentration >4.5 mmol/L (400 mg/dL) [14].

TGs are determined by enzymatic methods, usually after the release of glycerol in an enzymatic or alkaline hydrolysis reaction. The acceptable total error of a TG concentration determination recommended by the US National Cholesterol Education Program (NCEP) is ±15%, and the maximum allowable in the tests of the Central Research Center for Quality in Laboratory Diagnostics (COBJwDL) is ±10%. The concentration of TC in serum/plasma is determined by enzymatic methods using automatic analyzers. The acceptable total error in the determination of TC concentration recommended by the NCEP, is ±9%, and the value applicable to the COBJwDL testers is ±8%.

HDL-C concentration determinations are performed in serum or plasma. In the late 1990s, direct methods for the determination of HDL-C replaced precipitation methods. Homogeneous new-generation methods (several types) are widely available, and ready-to-use reagents enabled the full automation of the determination of the concentration of HDL in a primary serum/plasma sample. Direct methods are well standardized (for samples from healthy individuals) and show good precision of the determinations. Deviations in the results (measurement bias) are mainly due to the substrate (matrix), such as in dyslipidemia.

The reference method for determining LDL-C concentration is Beta quantification, which is based on preparative ultracentrifugation of the sample (serum, plasma), separating the lipoproteins according to their density into two fractions. In daily practice, LDL-C concentration is mostly calculated; direct (homogeneous) methods are used less frequently.

The Friedewald formula, using determined TC, HDL-C, and TG levels and the adopted TG-to-VLDL-C ratio, is widely used to calculate the LDL level: LDL-C = TC − HDL-C − TG/5 (mg/dL) or LDL-C = TC − HDL-C − TG/2.2 (mmol/L) [12].

### 1.4. Direct Methods for Determination of LDL-C

The NCEP recommendations made in 1995 stated that new methods for LDL-C measurement should be developed and that such methods should be capable of quantifying LDL-C directly because of limitations of the Friedewald method. Thus, the routine measurement of LDL-C should assess the range of lipoproteins included in the LDL-C fraction measured by β-quantitation. The postulated advantages of direct LDL-C measurement are the ability to determine LDL-C at high levels of TG, the ability to measure LDL-C in non-fasting samples, and the reduction of imprecision by taking a single measurement instead of a calculated value from three different results. There are several commercial homogeneous methods for LDL-C. They use a wide variety of surfactants, ionic polymers, and other components that either selectively prevent or enable the measurement of lipoproteins classes. Homogeneous reagents for direct measurement of LDL-C offer automation and improved precision. There have been many studies evaluating direct lipoprotein measurements [15,16].

Homogeneous direct methods of LDL-C assessments are based on several chemical reactions. Reactions in Roche diagnostics: (1) HDL-C, VLDL-C, CM are blocked by surfactant and sugar compounds; (2) LDL-C is solubilized by the enzymes and surfactants Cholestenone + H_2_O_2_; (3) H_2_O_2_ + color forming reagents + enzyme. Color Daiichi diagnostics applies other reactions: (1) Surfactant 1 + enzymes react only with HDL-C-C, VLDL-C-C, and CM-C; (2) Peroxides created by non-LDL-C are converted to colorless products; (3) LDL-C-C + surfactant 2 + enzymes + color forming reagents color. Denka-Seiken measurements are based on another sequence: (1) Non-LDL-C + surfactant combination 1 + enzymes cholesteonone + H_2_O_2_; (2) H_2_O_2_ + catalase H_2_O; (3) LDL-C-C + surfactant combination 2 cholestenone + H_2_O_2_ (4); Catalase inactivated by azide; (5) H_2_O_2_ + color forming reagent + enzymes color. However, Wako Labs follows another sequence: (1) LDL-C + Protecting reagent protects LDL-C from enzyme reactions; (2) HDL-C-C, VLDL-C-C, CM-C + enzymes H_2_O_2_ + catalase H_2_O; (3) LDL-C deprotecting agent; (4) LDL-C + enzymes Cholestenone + H_2_O_2_; (5) H_2_O_2_ + color forming reagents color [17].

### 1.5. The Friedewald Formula for Determination of LDL-C

The value of LDL-C lipoproteins can be calculated using the Friedewald formula (FF): LDL-C [mg/dL] = TC − TG/5-HDL-C (where all concentrations are given in mg/dL) or LDL-C [mmol/L] =TC − TG/2.2 (where all concentrations are given in mmol/L). The quotient TG/5 is usually used as an estimate of VLDL-C concentration. It assumes that virtually all of the plasma TG is carried in VLDL-C, and that the TG: cholesterol ratio of VLDL-C is constant at about 5 [18]. This formula does not apply if the triglyceride value exceeds 400 mg/dL, i.e., 4.6 mmol/L. However, it should be remembered that therapeutic algorithms for hypercholesterolemia in childhood are based on the LDL-C level determined in fasting samples, and the result obtained from the formula may be up to 25% error-biased [5,17]. Recently published Polish recommendations state that blood samples for routine lipid profile testing, primarily LDL-C and TC levels, do not have to be taken on an empty stomach. Repeated testing, due to the correctness of LDL cholesterol measurements in fasting samples, should be considered with non-fasting TG levels > 400 mg/dL (>4.5 mmol/L) [14].

The FF requires the measurements of TC, HDL-C, and TG to be in mg/dL. However, the estimations differ when calculated in mmol/L. The FF has been the standard method for LDL-C assessment because it is economical and simpler than direct assays and the most accurate LDL-C measurement methods. FF has limitations under certain conditions, especially when metabolic abnormalities alter the relationship between VLDL-C and TG, as in high hypertriglyceridemia (TG > 400 mg/dL). Moreover, studies have demonstrated considerable differences between the estimation and direct assessment of LDL-C concentrations in many other conditions [19].

The authors state that the method is an approximation assessment. It is not applicable in the presence of chylomicrons and in type 3 hyperlipoproteinemia. The equation has not been validated for use in patients on statins or other lipid lowering drugs, as it assumes a constant content of cholesterol in VLDL-C. Several researchers have worked to improve the Friedewald equation by adjusting the conversion factor. This led to the development of further modifications and novel equations for estimating LDL-C using adjustments for the TG/VLDL-C ratio. The Friedewald equation assumes that the concentration of VLDL-C is low compared to LDL-C. Reliable calculation with FF requires a fasting specimen obtained after at least a 12 h fast. The LDL-C fraction obtained by the FF includes both IDL and lipoprotein (a) (Lp(a)). Reports suggest an underestimation of LDL-C calculated by the FF at low LDL-C levels and high TG levels, ≥150 mg/dL [19].

A modified Friedewald formula was developed using the Martin–Hopkins equation: LDL-C = TC − HDL-C − TG/x (in mg/dL), where x is the ratio of TG to VLDL-C. Values are available in special tables or online calculators, e.g., www.LDL-calculator.com. This formula allows for a more accurate calculation than the Friedewald formula of the LDL-C concentration at low values and when the TG concentration is 2.0, TG concentration is 2.0–4.5 (175–400 mg/dL), including in non-fasting samples [20].

In the Martin–Hopkins equation, VLDL-C is calculated using a set of variables of TG divisors (180 factors) based on TG and non-HDL-C intervals, determined empirically by the vertical autoprofile (VAP) ultracentrifuge method. Because the TG/VLDL-C ratio for TG-rich lipoproteins (TRL) is continuous, it leads to either an underestimation or overestimation of VLDL-C at the ends of each factor interval [21].

### 1.6. The Sampson–NIH Equation to Calculate LDL-C

In 2020 The Sampson–NIH equation was presented: LDL-C = TC/0.948 − HDL-C/0.971 − (TG/8.56 + [TG × NonHDL-C]/2140 − TG^2^/16,100) − 9.44. It was developed by fitting to LDL-C values determined by the “gold-standard” β-quantification reference method. Thus, it allows for a smooth factor line between the Friedewald and Martin–Hopkins equations. The Sampson–NIH formula assumes that estimated LDL-C always changes in a continuous and predictable manner. Additionally, it is easy to implement in a standard laboratory information system [21,22].

The Sampson–NIH formula allows for accurate calculation of LDL-C concentration at its low values and very high TG concentrations—even up to 800 mg/dL (9.4 mmol/L) [(https://nih.figshare.com/articles/code/Equation_Calculator_for_Low_Density_Lipoprotein_Cholesterol/11903274) (accessed on 27 October 2025)]. It can be easily configured in laboratory IT systems or other types of software. Calculated LDL-C concentration is also burdened with several errors whose results are used in other formulas, hence the independent role of accuracy and precision of TC, HDL-C, and TG in these calculations [14].

The Academy of Pediatrics emphasizes that to obtain an accurate picture of lipid metabolism, tables taking the child’s age and gender into account should be used. Values above the 95th percentile for total cholesterol, triglycerides, LDL-C, and non-HDL-C fractions and below the 5th percentile for HDL-C should be considered abnormal. Ultimately, the plasma LDL-C concentration should not exceed 130 mg/dL. In children with obesity, diabetes, or metabolic syndrome, LDL-C levels should not exceed 110 mg/dL [23]. In patients with TG concentrations >200 mg/dL (>2.3 mmol/L), obesity, type 2 diabetes, metabolic syndrome, and LDL-C concentration <70 mg/dL (<1.8 mmol/L), it is recommended to calculate the concentration of non-HDL-C or determine the concentration of apoB in serum/plasma instead of LDL-C [14].

In line with the position of the European Atherosclerosis Society (EAS) (2019) and the European Federation of Clinical Chemistry and Laboratory Medicine (EFLM) (2016), it is best to use the LDL-C concentration determined by the homogeneous method. Most of the available data considers the cut-off point above which the risk of cardiovascular events increases significantly to be a concentration ≥25 mg/dL (≥0.6 mmol/L) (increase in the risk of heart attack by 33%), while others indicate a different cut-off point: 30–40 mg/dL (0.75–1.0 mmol/L) [14,24,25,26].

### 1.7. Reporting Lipid Panel

The lipid profile includes the set of tests performed in plasma or serum and calculations in order to diagnose dyslipidemia. A laboratory lipid profile report, in addition to the assay results, should include information on how the LDL-C concentration was determined (calculated/measured) and the target concentrations of the assayed analytes. In severe dyslipidemia, a report should also include information on the urgency of seeing a doctor in cases of LDL-C concentration indicating a possible diagnosis of heterozygous (>190 mg/dL, 5.0 mmol/L) or homozygous (>500 mg/dL, 13.0 mmol/L) hyper familial chlesterolemia (fH), Lp(a) levels > 180 mg/dL (450 nmol/L), indicating a very high risk of cardiovascular incidents, or a TG concentration >880 mg/dL (10.0 mmol/L) indicating a high risk of acute pancreatitis or familial chylomicronemia syndrome (FCS) [12].

These methods are used in automatic analyzers. Direct methods of LDL-C assessment vary in terms of accuracy due to significant methodological diversity. The permissible total error in determining/calculating LDL-C concentration, as recommended by NCEP, is ±12%) [12].

The purpose of this study was to present the analysis of lipid panel results in pediatric patients. We wanted to demonstrate differences in the levels of cholesterol parameters and TG in the age groups. We focused on comparisons between the three methods used to assess LDL-C, i.e., direct assay, the Friedewald formula, and the Sampson–NIH Formula, to check whether they differed statistically. Based on the results, we would attempt to validate the formulas for pediatric patients.

## 2. Material and Methods

The material for study comprised the results of blood tests of in-patients performed at the University Pediatric Hospital Laboratory, Lublin, Poland in the period from April 2023 to February 2024. All children with obesity, suspected of thyroid disorders, diabetes, cardiac diseases, and nephrotic syndromes, family history of hypercholesterolemia, gastric disorders, and parenteral nutrition undergo a routine lipid panel test. The study was conducted in a large group of in-patients aged 8 months to 18 years. In total, 3000 result reports were analyzed. The patients who did not have all lipid parameters measured were excluded. Finally, 1982 reports were considered for further analysis. Lipid profile TC, TG, HDL-C, and non-HDL-C were measured with an automated analyzer Cobas Roche 501. LDL-C was calculated by the direct assessment method, the Friedewald formula, and the Sampson–NIH equation. The protocol of the study was approved by the University Bioethics Committee KE-0254/189/10/2022 of 6 October 2022.

### 2.1. Statistical Analysis

In the conducted study, the level of statistical significance was assumed at α = 0.05, which means accepting the risk of making a type I error at the level of 5%. The Shapiro–Wilk test was used to assess the normality of the data distribution.

Distributions of numerical variables are presented in the form of the median (Mdn) as a measure of central tendency, preferring this indicator due to its robustness to outliers and nonparametric nature. In addition, the values of the first (Q1) and third quartiles (Q3) are presented, which allows us to define the range in which 50% of the observations from the sample are concentrated.

For categorical variables, the numbers (*n*) and percentages of individual categories were presented, which enabled the assessment of their distribution in the sample. When comparing measures of central tendency between two independent groups for a continuous variable with a non-normal distribution, the nonparametric Wilcoxon rank sum test was used. Differences between categorical variables were assessed using the Pearson chi-square test, which allowed for the analysis of relationships between categories. Agreement between pairs of competing methods in estimating LDL concentration was assessed and visualized using Bland–Altman analysis (also known as Tukey’s mean difference plot) [24,27].

Multivariate effects analysis was performed using a mixed linear regression model with a robust linear mixed model (RLMM) [25]. A mixed model including a random effect was fitted using the DAStau method. Differences in LDL levels between groups were compared using contrast analysis based on estimated marginal means (EMM) across subgroups. *P* values and 95% confidence intervals (95% CI) were estimated based on the asymptotic approximation of the Wald test.

### 2.2. Characteristics of the Statistical Tool

Analyses were performed using the R statistical language (version 4.3.3) [26] on Windows 11 Pro 64 bit (build 22631), using the packages lme4 (version 1.1.35.2) [28], Matrix (version 1.6.5) [29], bland (version 0.6.0) [30], robustlmm (version 3.3.1) [25], emmeans (version 1.10.1) [31], sjPlot (version 2.8.15) [32], performance (version 0.12.3) [33], report (version 0.5.8) [34], gtsummary (version 1.7.2) [35], ggplot2 (version 3.5.0) [36], and dplyr (version 1.1.4) [37].

## 3. Results

### 3.1. Sample Characteristics

The results of 1982 pediatric patients aged 1 week to 17 years who underwent lipid profile assessment were analyzed. The sample was balanced in terms of sex, with slightly more boys than girls. The study group included 960 girls (48.44%) and 1022 boys (51.56%). This relatively even representation of both sexes is beneficial from the analysis perspective, as it allows for more reliable intergroup comparisons. The age distribution in the sample overall and by sex is presented in Table 1.

In the whole cohort, the median age was 13 years, with a quartile range (Q1, Q3) of 9 to 15 years. The age in this sample shows some variability, but most patients are in school age and early adolescence. When stratified by gender, some differences in the median age were observed between boys and girls. The median age of boys was 12 years (Q1 = 9, Q3 = 15), while that of girls was 13 years (Q1 = 9, Q3 = 15). The statistical significance of *p* = 0.011 suggests that boys were significantly younger than girls in the sample.

The division into age groups by median (<13 years vs. ≥13 years) also reveals interesting differences. In the group of subjects younger than 13 years, there are slightly more boys (52.29%) than girls (47.55%), while in the group ≥ 13 years the proportion is reversed —a larger percentage of girls (52.45%) is seen. A significant test result (*p* = 0.035) indicates the significance of the differences.

### 3.2. Characteristics of Lipid Profile Assessment of the Tested Sample

In this analysis, the lipid profile of the study sample was assessed, taking into account differences between boys and girls. Table 2 presents detailed results of lipid assessment in the total sample and by sex, along with a statistical assessment of the significance of differences between the two groups.

Lipid profile assessment results in the pediatric study sample showed significant differences in some lipid parameters between boys and girls. The median triglyceride concentration in the total sample was 81 mg/dL, with quartile values ranging from 60 to 111 mg/dL. When stratified by sex, boys had slightly higher triglyceride concentrations—83 mg/dL (61–113 mg/dL)—while in girls the median was 79.5 mg/dL (59–110 mg/dL), although this difference was not statistically significant (*p* = 0.054).

The differences were more pronounced for total cholesterol. The median total cholesterol in the overall sample was 153 mg/dL (135–171 mg/dL), boys having higher values—154 mg/dL (137–172 mg/dL)—compared to 150 mg/dL (134–169 mg/dL) in girls. This difference was statistically significant (*p* = 0.009) suggesting that boys may have higher total cholesterol levels than girls.

A similar trend was observed for HDL cholesterol. In the overall sample, the median was 54 mg/dL (45–64 mg/dL), boys having slightly higher values (55 mg/dL, 46–64 mg/dL) than girls (54 mg/dL, 44–64 mg/dL). This difference was also statistically significant (*p* = 0.043), suggesting a small but noticeable difference in HDL levels between the sexes.

The values of non-HDL cholesterol and LDL-D (direct method) did not show significant differences between sexes. The median non-HDL cholesterol in the total sample was 97 mg/dL (80–115 mg/dL), with values of 99 mg/dL (81–115 mg/dL) in boys and 97 mg/dL (79–114 mg/dL) in girls (*p* = 0.100). For LDL-D, the median in the total sample was 95 mg/dL (79–112 mg/dL), with values of 97 mg/dL (79–112.25 mg/dL) in boys and 94 mg/dL (78–112 mg/dL) in girls (*p* = 0.137).

LDL-C analyses using both the Friedewald and Sampson–NIH formulas did not reveal significant differences between the sexes. The median LDL-C indicated by the Friedewald formula was 78.60 mg/dL (63–94.75 mg/dL) in the overall sample, with values of 79.60 mg/dL (63.40–95.25 mg/dL) in boys and 77.80 mg/dL (62.60–94.55 mg/dL) in girls (*p* = 0.294). The Sampson–NIH formula yielded similar results—the median in the overall sample was 79.72 mg/dL (64.17–96.51 mg/dL) with values of 80.71 mg/dL (64.50–96.82 mg/dL) in boys and 78.74 mg/dL (63.57–96.47 mg/dL) in girls (*p* = 0.266).

This analysis also assessed dyslipidemia divided into two age groups: patients under 13 years of age and patients aged 13 years and older (Table 3).

TG levels were comparable between age groups, with a median of 81 mg/dL (59–114 mg/dL) in the entire sample, a median of 81.50 mg/dL (60–108 mg/dL) in the <13-year-old group, and 81 mg/dL in the ≥13-year-old group (*p* = 0.816), indicating no statistically significant differences in triglyceride levels between age groups.

In the case of total cholesterol, a clear difference was observed. In the group <13 years, the median was 157 mg/dL (138–174 mg/dL), while in the group ≥13 years it was significantly lower—149 mg/dL (131.25–167.75 mg/dL). This difference was statistically significant (*p* < 0.001), suggesting that the younger age group is characterized by higher total cholesterol levels.

HDL cholesterol levels also differed between age groups. Patients <13 years had a median of 55 mg/dL (46–65 mg/dL), whereas in the older group the median was 53 mg/dL (45–63 mg/dL). This difference was statistically significant (*p* = 0.044), suggesting lower HDL cholesterol values in older patients.

Non-HDL cholesterol showed a similar trend to total cholesterol. In the <13 years group, the median was 100 mg/dL (83–117 mg/dL), while in the ≥13 years group it was significantly lower—93 mg/dL (77–112 mg/dL), with *p* < 0.001. These results indicate higher non-HDL cholesterol levels in younger patients.

In the case of direct LDL-D measurement, patients <13 years of age had higher concentrations—median 97 mg/dL (81–115 mg/dL) compared to 93 mg/dL (76–110 mg/dL), in the group ≥13 years of age. This also turned out to be statistically significant (*p* < 0.001).

### 3.3. Estimation of the Agreement of LDL Results Assessed by Direct Method vs. LDL-C Calculated by the Friedewald Formula

**Figure 1:**
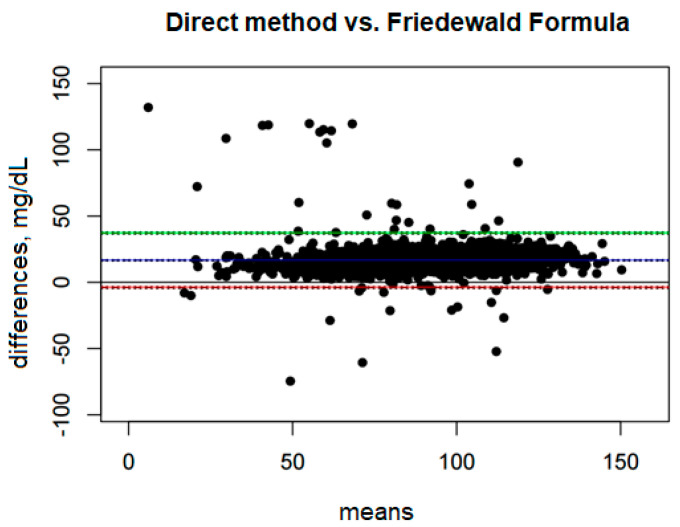
Blandt–Altman plot comparing the agreement of LDL test results between the direct method and the Friedewald formula. (The points on the plot represent the differences for individual patients between the direct method and the Friedewald formula, referred to the mean values obtained from both methods. A single point represents the difference for a single patient, allowing an assessment of how large these differences are depending on the mean LDL level for each person).

The mean bias is 16.61 mg/dL, which means that the Friedewald formula systematically underestimates LDL values compared with the direct method (Figure 1). The confidence interval for this bias, ranging from 16.15 mg/dL to 17.07 mg/dL, is narrow, indicating a precise estimation of this bias.

The Upper Limit of Agreement (ULoA) is 37.14 mg/dL, which means that in extreme cases the differences between the methods can be as much as 37.14 mg/dL in favor of the direct method. In turn, the Lower Limit of Agreement (LLoA) is −3.92 mg/dL, which indicates that in some cases the Friedewald formula may overestimate LDL-D compared with the direct method, although these overestimates are much rarer and smaller than underestimates.

The range between the lower and upper limits of agreement is 41.07 mg/dL, suggesting that the variability of the differences between these methods is quite large. The bias constitutes as much as 40.45% of this range, indicating a significant systematic deviation between the methods.

### 3.4. LDL-D Measured by Direct Method vs. LDL-C Calculated by the Sampson–NIH Formula

**Figure 2:**
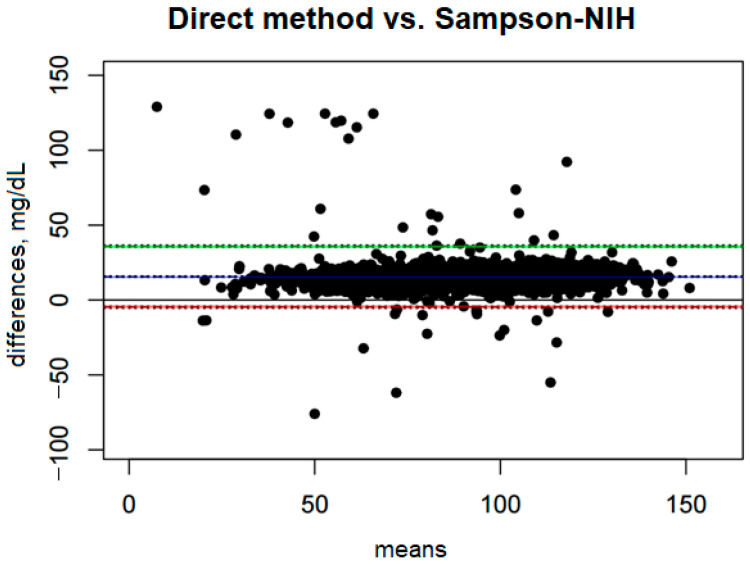
Blandt–Altman plot comparing the agreement of LDL test results between the direct method and the Sampson–NIH formula.

Comparison of the direct method with the Sampson–NIH formula (Figure 2) shows a systematic bias of 15.60 mg/dL, indicating that the Sampson–NIH formula underestimates LDL values compared to the direct method. The confidence interval for this bias is narrow (from 15.15 to 16.06 mg/dL), suggesting that the estimation of the difference is quite precise.

The ULoA is 35.92 mg/dL, and the LLoA is −4.72 mg/dL. These results suggest that in most cases the differences between the two methods fall within this range, meaning that the Sampson–NIH formula may slightly overestimate or underestimate results, although underestimations are much more frequent and significant.

The range between the lower and upper limits of agreement is 40.64 mg/dL, indicating moderate variability in the differences between methods. Bias accounts for 38.39% of this range, indicating that systematic bias has a significant impact on the differences between methods.

### 3.5. LDL-C, Calculated by the Friedewald Formula vs. LDL-C and the Sampson–NIH Formula

**Figure 3:**
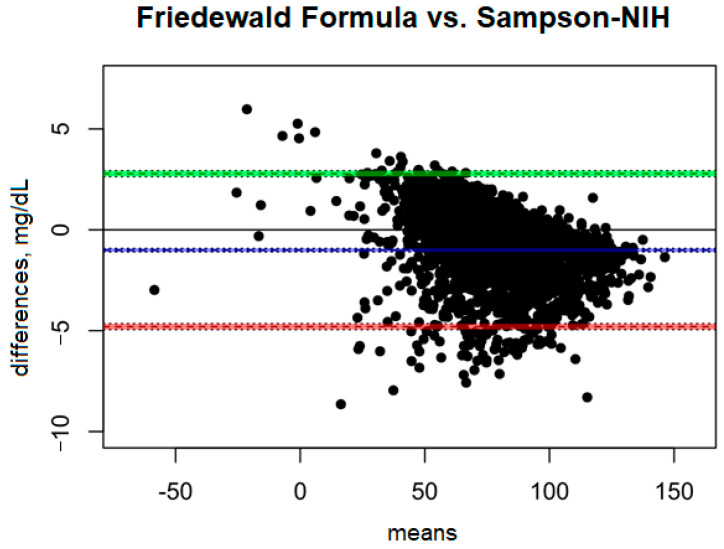
Blandt–Altman plot comparing the agreement of LDL test results between the Friedewald formula and the Sampson–NIH formula.

A comparison of the Friedewald formula with the Sampson–NIH formula (Figure 3) shows very small differences between the two methods. The mean bias is only −1.01 mg/dL, suggesting that the Sampson–NIH formula gives slightly higher results than the Friedewald formula, though the difference is minimal. The confidence interval for this bias (−1.09 to −0.92 mg/dL) is very narrow, indicating a high precision of the difference estimate.

The ULoA is 2.78 mg/dL and the LLoA is −4.80 mg/dL, indicating that in most cases the differences between the two methods are within this range, with the Sampson–NIH results predominating. The range of differences between the methods is small (7.58 mg/dL), indicating good agreement.

### 3.6. Estimation of the Effects of Age and Sex on LDL Scores in a Multivariate Approach

#### 3.6.1. Selection of Variables and Effects for a Multifactor Model

Variables were selected for the model using the stepwise addition method, starting with a model containing only a constant and a random intercept for a patient. In the first step, the baseline model was compared with the model containing the method explanatory variable. Models were compared based on AIC and BIC and χ^2^ statistics, with lower AIC and BIC values indicating better model fit (considering complexity). In the next steps, age and sex variables were added, which gradually improved the model’s fit. Next, the effects of interactions between variables were tested. The interaction between method and age significantly improved the model fit (*p* < 0.001), while interactions with gender, including the triple interaction, did not show significant improvement (*p* > 0.05). The final model included method, age, gender, and the interaction between the method and age variables, with an additive effect of sex (see Appendix A for specifications).

#### 3.6.2. Characteristics of the Overall Fit of the Model

The regression model took into account the repeatable study design resulting from the numerous repeated measurements of the LDL parameter using three competing methods. Therefore, the model was fitted to 5946 observations taken of 1982 unique individuals (id), each with three repeated measurements. Factors such as sex and age were kept constant for each subject, regardless of the LDL measurement method.

For most observations, the residuals are close to zero (Mdn = 0.012) and the quartile values of the residuals were characterized by symmetric results (Q1 = −0.500, Q3 = 0.512), suggesting that in the vast majority of cases the model predicts the outcomes well.

Most observations (5114 of 5946) had robust weights close to 1.0, suggesting that these observations have minimal impact on the model fit and present a good fit. The remaining 832 observations have weights of less than 1.0, indicating that their impact on the model is limited. The median weight for these observations is 0.687, and the minimum weight is 0.03, indicating that some observations are significantly attenuated in the model fit, probably due to extreme values of the residuals.

Similarly, in the case of the random effects, 1589 of the 1982 units have weights close to 1.0, indicating good fit of the data to the model for most units. The remaining 393 units have reduced weights, with a median of 0.866, indicating that these units had less influence on the model. The lowest weight is 0.232, indicating units that were significantly impaired in the model fit, probably due to high inter-unit variability.

For random effects, the variance of the constant is τ00 and d = 554.84, with an SD of 23.56, which indicates a significant inter-unit variability (id). The variance of the residuals is σ^2^ = 6.33, with a standard deviation of 2.52, which suggests that the intra-unit variability of the LDL parameter is relatively low compared to the inter-unit variability.

The intraclass correlation coefficient (ICC) value = 0.99 indicates that as much as 99% of the total variability in the data can be attributed to differences between patients (id), and only 1% of the variability is due to differences within individuals. This high ICC value suggests that the model is good at isolating differences between groups.

The marginal R^2^ is 0.095, which means that the fixed effects in the model (independent variables) account for app. 9.5% of the variability in the data. The conditional R^2^ is 0.990, which indicates that the entire model, including random effects, explains as much as 99% of the variability, confirming that random effects are crucial in this model.

#### 3.6.3. Characteristics of Model Coefficients

Based on the results of the RLLM model analysis (Table 4), the mean LDL level for the group of girls aged 13 years measured using the direct method was 95.18 mg/dL (95% CI: 93.59–96.77, *p* < 0.001).

The coefficient values for the Friedewald method (β = −16.27 mg/dL, 95% CI: −16.44–−16.10, *p* < 0.001) and the Sampson–NIH method (β = −15.33 mg/dL, 95% CI: −15.50–−15.16, *p* < 0.001) are very similar, suggesting that both methods better determine lower LDL concentrations than the direct method, with the underestimation by the Friedewald formula being slightly greater.

Age also showed a significant association with LDL levels (β = −0.37 mg/dL, 95% CI: −0.63–−0.12, *p* = 0.004). The negative coefficient value indicates that LDL levels decrease with increasing age, with age centered at the median of 13 years.

Interactions between measurement methods and age indicate an additional effect of age on LDL assessments depending on the method used. Both the Friedewald method (β = −0.12 mg/dL, 95% CI: −0.16–−0.08, *p* < 0.001) and the Sampson–NIH formula (β = −0.15 mg/dL, 95% CI: −0.18–−0.11, *p* < 0.001) show significant interactions with age, suggesting that the differences between these methods and the direct method become greater with increasing age. This means that in older patients, the underestimation of LDL levels using the Friedewald formula and especially the Sampson–NIH formula may be more pronounced.

### 3.7. EMM Results: Contrast Analysis

According to the data in Table 5, the direct measurement method predicts the highest LDL value (95.1 mg/dL) for the age of 13 years, which is significantly different from the results obtained by the Friedewald method (79.0 mg/dL) and Sampson–NIH (80.0 mg/dL).

The contrast analysis shown in Table 6 confirms that the difference between the direct method and the Friedewald method is 16.10 mg/dL, and between the direct method and the Sampson–NIH is 15.12 mg/dL, which indicates a systematic underestimation of LDL concentrations by the indirect methods. The z values for both contrasts are very high, which in combination with the *p*_adj_ values < 0.001 clearly confirms the significance of the differences.

The contrast value between the Friedewald and the Sampson–NIH methods is −0.98 mg/dL, which indicates that the differences between the two methods are much smaller, although still statistically significant (*p*_adj_ < 0.001). This result suggests that although both indirect methods underestimate LDL levels compared to the direct method, the differences between them are small but still detectable.

**Figure 4:**
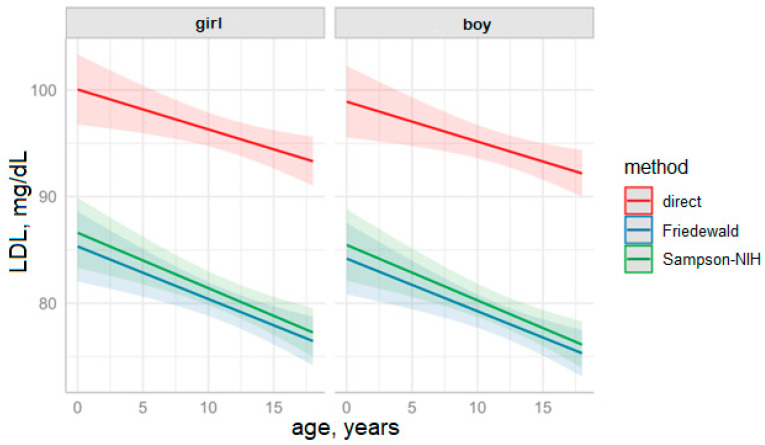
Predictions of LDL values based on the fitted LDL model over the years by measurement method and sex (solid lines represent means, colored background indicates 95% CI.

The visualization of the estimated marginal means with predictions for the entire range of age values with reference to testing method and patient sex is presented in Figure 4.

### 3.8. Comparative Evaluation of Indirect Methods for Dyslipidemia Diagnosis Against Direct LDL Measurement

We evaluated the diagnostic performance of two indirect methods—the Friedewald equation and the Sampson–NIH equation—for identifying dyslipidemia in children aged over 2 years, using direct LDL cholesterol (LDL-C) measurement as the reference standard. Dyslipidemia was defined as LDL-C >100 mg/dL (2.6 mmol/L) and/or elevated triglyceride (TG) levels, with thresholds of ≥100 mg/dL (≥1.1 mmol/L) for children under 10 years and ≥130 mg/dL (≥1.5 mmol/L) for those aged 10–18 years. The dataset comprised 1939 samples for the pediatric subgroup 2–18 years, with noted actual dyslipidemia diagnosis of 50.3% (*n* = 975).

### 3.9. Analysis of Incorrect and Missed Diagnoses

For the Friedewald equation, 351 incorrect diagnoses were observed: 3 false positives (non-dyslipidemic children incorrectly classified as having dyslipidemia) and 348 false negatives (children with dyslipidemia missed by the method). This represents a total misclassification rate of 18.1% (351/1939). The Sampson–NIH equation resulted in 333 incorrect diagnoses: 3 false positives and 330 false negatives, yielding a slightly lower misclassification rate of 17.2% (333/1939). The reduction in false negatives by 18 cases with the Sampson–NIH method reflects its improved sensitivity, identifying 645 true positives compared to 627 for Friedewald.

The confusion matrices in Table 7 and Table 8 summarize the classifications for each indirect method, where rows represent predictions by the indirect method and columns represent true outcomes by the direct method.

Table 9 presents a side-by-side comparison of essential metrics for both methods, facilitating direct assessment of their strengths and limitations.

## 4. Discussion

The results of the Lipid Panel Test are pivotal to plan a patient’s healthcare. Ideally, total cholesterol should be less than 200 mg/dL. Higher levels might indicate an increased risk of heart disease. The LDL-C levels above 160 mg/dL are considered high and can lead to plaque build-up in the arteries, increasing the risk of atherosclerosis. Higher levels of HDL-C, preferably 60 mg/dL or above, are desirable. Triglycerides values should be below 150 mg/dL. Elevated levels can indicate metabolic syndromes like diabetes. Although there is insufficient evidence to recommend for or against screening in young patients without signs or symptoms, a serum lipid panel is the most commonly proposed screening test for FH and multifactorial dyslipidemia [38].

The evolving role of the clinical laboratory is to provide more information to clinicians to assess cardiovascular disease (CVD) risk and implement effective therapy. However, routine methods used to determine LDL-C-cholesterol (LDL-C-C), like the Friedewald formula, ultracentrifugation, electrophoresis, and homogeneous direct methods have certain limitations. Studies suggest that LDL-C and HDL-C size or particle concentration are alternative methods to predict future CVD risk. At present, there is no consensus role for lipoprotein particle or subclasses in CVD risk assessment. LDL-C and HDL-C particle concentrations are measured by several methods, e.g., gradient gel electrophoresis, ultracentrifugation-vertical auto profile, nuclear magnetic resonance, and ion mobility. It has been suggested that HDL-C functional assays may be better predictors of CVD risk [17].

The present study assessed dyslipidemia in two age groups <13 years and ≥13 years. TG levels were comparable between the age groups, with a median of 81 mg/dL (59–114 mg/dL) in the entire sample. The measurements of TC found statistically significant (*p* < 0.001) differences between the groups, suggesting that the younger patients had higher TC levels. Also, the levels of HDL were statistically different (*p* = 0.044), suggesting lower HDL cholesterol values in older patients and higher non-HDL levels in younger patients. Direct LDL-D measurement revealed that patients <13 years of age had higher concentrations compared to the group ≥13 years of age (*p* < 0.001).

The methods to assess LDL-C measurements were reviewed in search of the best one. In total, the reports identified 45 studies that compared various formulae and listed several limitations of each equation. The Martin/Hopkins equation uses an adjustable factor for TG—very low-density lipoprotein-cholesterol ratios—and yields more a accurate LDL-C-C calculation, especially when LDL-C < 1.81 mmol/L (<70 mg/dL) and TG is elevated. The Sampson equation was elaborated for TG up to 9 mmol/L (800 mg/dL) and was based on β-quantification; it performs well on high TG postprandially and low LDL-C-C samples, similarly to direct LDL-C-C. The Friedewald formula has several recognized limitations, especially inaccurate results for triglycerides (TG) > 4.5 mmol/L (>400 mg/dL). We concluded that the choice of equation should consider the level of triglycerides [39].

We compared LDL-C values calculated by the direct method, the Friedewald formula, and the Sampson–NIH Formula in a large group of pediatric patients (1982) aged 8 months to 18 years, 960 girls (48.44%) and 1022 boys (51.56%). The analysis of LDL-C using the Friedewald and Sampson–NIH formulas yielded similar results, with higher median LDL-C values in the younger group (<13 years), 81.90 mg/dL (65.40–97.20 mg/dL) by the Friedewald formula and 82.78 mg/dL (66.63–98.45 mg/dL) by the Sampson–NIH formula, compared to medians of 76.40 mg/dL (60.65–92.60 mg/dL) and 76.74 mg/dL (60.68–93.98 mg/dL) in the ≥13 years group, respectively. Both differences were statistically significant (*p* < 0.001).

Another study on a sample of 135 individuals reported that the statistical analysis of LDL-C calculated by FF showed a mean value of 124.4 mg/dL (SD = 42.1 mg/dL); measured by direct assay, the mean value was 125.1 mg/dL (SD = 38.5 mg/dL). It showed strong correlation between the methods, as the mean difference was −0.7 mg/dL. It was concluded that there is no significant difference between the assessment of LDL-C using the FF and by direct measurement. The researchers also noticed the FF underestimates the value of LDL-C compared to direct measurement. Moreover, the average difference between these methods appears to be more pronounced when the LDL-C is lower (by direct measurement): when LDL-C ≤ 121 mg/dL, the mean difference was 0.26 mg/dL and for LDL-C > 121 mg/dL, the mean difference was −1.62 mg/dL. However, despite being approximately six times greater when the absolute difference in LDL-C > 121 mg/dL, this result is still too small and is clinically insignificant [19].

The comparison between the direct method and the Sampson–NIH formula shows that the Sampson–NIH formula underestimates LDL values compared to the direct method. From a clinical perspective, the Sampson–NIH formula does not seem to be sufficiently consistent with the direct method for routine use as a substitute for LDL measurement, especially in cases where LDL measurement accuracy is critical. Although the differences are not as large as with the previous formula, the consistent underestimation of the results may lead to an underestimation of the lipid profile of patients, which may have a negative impact on therapeutic decisions. The analysis shows very small differences between the two methods. From a clinical point of view, the Friedewald and Sampson–NIH formulas can be considered interchangeable. The differences between them are small and probably do not have a significant impact on therapeutic decisions. Both methods can therefore be used interchangeably in the standard assessment of LDL-C without concerns about significant differences in the results.

However, the analysis found that LDL levels differed significantly between the measurement methods. When the direct method was used as the reference category, the Friedewald method and the Sampson–NIH formula systematically underestimated LDL values compared to the direct method. For clinicians, this means that interpretations of LDL results obtained using these formulas may lead to an underestimation of cardiovascular risk, especially when the results are compared with reference values based on the direct method. In clinical practice, this means that for patients with borderline or low LDL values, the use of the Friedewald or Sampson–NIH method may lead to treatment decisions that do not fully reflect the actual risk.

Both the Friedewald and Sampson–NIH equations demonstrate high specificity (99.7%), ensuring minimal false positives (only three cases each), which reduces unnecessary interventions and supports efficient resource use in pediatric settings. Their positive predictive values (99.5%) confirm that positive results are highly reliable, making these methods valuable for confirming dyslipidemia in children with clinical indications, such as a family history of cardiovascular disease or obesity.

However, the moderate sensitivity—64.3% for Friedewald and 66.2% for Sampson–NIH—reveals a significant limitation: a substantial number of dyslipidemia cases are missed (348 and 330 false negatives, respectively). This systematic underestimation of LDL-C concentrations results in detection of prevalence (32.5% for Friedewald, 33.4% for Sampson–NIH) well below the true prevalence of 50.3%. In clinical practice, this underestimation could delay the identification of children at risk for early cardiovascular issues, particularly those with elevated LDL-C (>100 mg/dL) or triglycerides (≥100 mg/dL for <10 years, ≥130 mg/dL for 10–18 years). Missed diagnoses are particularly concerning in pediatrics, where early intervention through lifestyle changes or in severe cases pharmacotherapy can mitigate long-term risks.

The Sampson–NIH method’s slight advantage in sensitivity (1.84% higher) and negative predictive value (1.03% higher) translates to 18 fewer missed cases, which is clinically meaningful in screening contexts, especially for children with elevated triglycerides, where the Sampson–NIH equation is optimized. The kappa statistics (0.639 for Friedewald, 0.657 for Sampson–NIH) indicate moderate to substantial agreement with the direct method, with Sampson–NIH showing better concordance. Balanced accuracies (82–83%) reflect good overall performance, though McNemar’s test highlights a predominance of false negatives, underscoring the need for caution with negative results.

In clinical practice, the systematic underestimation by both methods indicates they are best suited as initial screening tools rather than definitive diagnostics. The high number of missed diagnoses (17.9% of the sample for Friedewald, 17.0% for Sampson–NIH) indicates that direct LDL-C measurement should be employed to confirm negative results, particularly in high-risk children. The Sampson–NIH method’s improved detection makes it preferable for screening, but neither method fully replaces direct measurement for accurate lipid management in pediatric dyslipidemia.

Interactions between measurement methods and age indicate an additional effect of age on LDL assessments depending on the method used. We observed significant interactions with age, suggesting that the differences between these methods and the direct method become greater with increasing age. This means that in older patients, the underestimation of LDL levels using the Friedewald formula and especially the Sampson–NIH formula may be more pronounced. In clinical practice, especially in older patients, special attention should be paid to the choice of LDL measurement method to avoid underestimations of LDL cholesterol levels, which could lead to delay or inappropriate intensity of therapy.

In terms of sex, the lack of significant differences in LDL levels between boys and girls suggests that sex should not be a decisive factor in the selection of the method to measure LDL in this age group. Therefore, clinical decisions regarding the assessment of cardiovascular risk should not be significantly differentiated based on gender in the analyzed population of children and adolescents.

## 5. Conclusions

The analyses allowed for formulating the following conclusions:

1. The direct measurement method yields the highest LDL values for the age of 13 years, which is significantly different from the results obtained by the Friedewald method and Sampson–NIH equation.
2. The comparison between the direct method and Friedewald or Sampson–NIH method indicates a systematic underestimation of LDL concentrations by the indirect methods.
3. The comparison between the Friedewald and Sampson–NIH methods indicates small although still statistically significant differences. This suggests that although both indirect methods underestimate LDL levels compared to the direct method, the differences between them are small but still detectable.
4. From a clinical point of view, the Friedewald and Sampson–NIH formulas seem to be essentially interchangeable.

## Figures and Tables

**Table 1:** Characteristics of the sociodemographic profile of patients in the total sample and stratified by gender.

*p* ^c^	Sex		Sample (Total)	*n*	Characteristics
	Girl *n*_2_ = 1022 ^a^	Boy *n*_1_ = 960 ^a^			
**0.011**	13.00	12.00	13.00	1.982	Age (years)
	(1.00, 9.00)	(9.00, 15.00)	(9.00, 15.00)		
**0.035**				1.982	Age group (years):
	486.00 (47.55%)	502.00 (52.29%)	988.00 (4.85%)		<13 years
	536.00 (52.45%)	458.00 (47.71%)	994.00 (50.15%)		≥13 years

^a^ Mdn (Q1, Q3); ^c^ Wilcoxon Test.

**Table 2:** Lipid profile assessment results in the pediatric sample overall and stratified by sex.

*p* ^b^	Sex		Sample (in Total) ^a^	*n*	Characteristics
	Girl *n*_2_ = 1022 ^a^	Boy *n*_1_ = 960 ^a^			
0.054	79.50	83.00	81.00	1.982	TG [mg/dL]
	(59.00, 110.00)	(61.00, 113.00)	(60.00, 111.00)		
**0.009**	150.00	154.00	153.00	1.982	TC [mg/dL]
	(134.00, 169.00)	(137.00, 172.00)	(135.00, 171.00)		
**0.043**	54.00	55.00	54.00	1.982	HDL [mg/dL]
	(44.00, 64.00)	(46.00, 64.00)	(45.00, 64.00)		
0.1	97.00	99.00	97.00	1.981	non-HDL [mg/dL]
	(79.00, 114.00)	(81.00, 115.00)	(80.00, 115.00)		
0.137	94.00	97.00	95.00	1.982	LDL-D [mg/dL] direct method
	(78.00, 112.00)	(79.00, 112.25)	(79.00, 112.00)		
0.294	77.80	79.60	78.60	1.982	LDL-C, Friedewald Formula
	(62.60, 94.55)	(63.40, 95.25)	(63.00, 94.75)		
0.266	78.74	80.71	79.72	1.982	LDL-C, Sampson–NIH Formula
	(63.57, 96.47)	(64.50, 96.82)	(64.17, 96.51)		

^a^ Mdn (Q1, Q3); ^b^ Wilxocon Test.

**Table 3:** Lipid profile assessment results stratified by age group.

*p* ^b^	Age Groups		*n*	Characteristics
	≥13 Years	<13 Years		
	*n*_2_ = 994 ^a^	*n*_1_ = 988 ^a^		
0.816	81.50	81.00	1.982	TG [mg/dL]
	(60.00, 108.00)	(59.00, 114.00)		
**<0.001**	149.00	157.00	1.982	TC [mg/dL]
	(131.25, 167.75)	(138.00, 174.00)		
**0.044**	53.00	55.00	1.982	HDL [mg/dL]
	(45.00, 63.00)	(46.00, 65.00)		
**<0.001**	93.00	100.00	1.982	non-HDL [mg/dL]
	(77.00, 112.00)	(83.00, 117.00)		
**<0.001**	93.00	97.00	1.982	LDL-D [mg/dL] direct method
	(76.00, 110.00)	(81.00, 115.00)		
**<0.001**	76.40	81.90	1.982	LDL-C, Friedewald Formula
	(60.65, 92.60)	(65.40, 97.20)		
**<0.001**	76.74	82.78	1.982	LDL-C, Sampson–NIH Formula
	(60.68, 93.98)	(66.63, 98.45)		

^a^ Mdn (Q1, Q3); ^b^ Wilxocon Test.

**Table 4:** RLLM model coefficients.

LDL mg/dL			Explanatory Variables
*p*	95% CI	β	
**<0.001**	93.59–96.77	95.18	Constant
Reference category			Direct method
**<0.001**	−16.44–−16.10	−16.27	Friedewald Formula
**<0.001**	−15.50–−15.16	−15.33	Sampson–NIH Formula
**0.004**	−0.63–−0.12	−0.37	Age (centered for Mdn = 13.0 years)
Reference category			Sex [girl]
0.295	−3.28–0.99	−1.14	Sex [boy]
**<0.001**	−0.16–−0.08	−0.12	Method: [Friedewald] × age (centered for Mdn = 13.0 lat)
**<0.001**	−0.18–−0.11	−0.15	Method [Sampson–NIH] × age (centered for Mdn = 13.0 years)

**Table 5:** Estimated marginal means of LDL with reference to measurement method at age 13.0 years adjusted for gender.

Method	EMM	SE	95% CI
Direct	95.1	0.55	94.1–96.2
Friedewald	79.0	0.55	78.0–80.1
Sampson–NIH	80.0	0.55	78.9–81.1

**Table 6:** Results of the analysis of LDL concentration contrasts between measurement methods at age 13.0 years adjusted for sex.

Contrast	Estimate	SE	z	*p*_adj_
Direct	16.10	0.08	196.34	**<0.001**
Friedewald	15.12	0.08	184.43	**<0.001**
Sampson–NIH	−0.98	0.08	−11.91	**<0.001**

**Table 7:** Classification results for the Friedewald equation.

	True Dyslipidemia	True No Dyslipidemia
Dyslipidemia	627	3
No dyslipidemia	348	961

**Table 8:** Classification results for the Sampson–NIH equation.

	True Dyslipidemia	True No Dyslipidemia
Dyslipidemia	645	3
No dyslipidemia	330	961

**Table 9:** Performance metrics for Friedewald and Sampson–NIH equations in pediatric dyslipidemia diagnosis.

Classification Metric	Friedewald Equation	Sampson–NIH Equation	Notes
Accuracy (95% CI)	0.82 (0.80–0.84)	0.83 (0.81–0.84)	Proportion of correct classifications (true positives + true negatives)/total cases.
Kappa Statistic	0.639	0.657	Measure of agreement beyond chance, with higher values indicating better concordance.
Sensitivity	0.643	0.662	Proportion of true dyslipidemia cases correctly identified (true positives/actual positives).
Specificity	0.997	0.997	Proportion of non-dyslipidemia cases correctly identified (true negatives/actual negatives).
Positive Predictive Value (PPV)	0.995	0.995	Probability that a positive prediction is correct (true positives/predicted positives).
Negative Predictive Value (NPV)	0.734	0.744	Probability that a negative prediction is correct (true negatives/predicted negatives).
Detection Rate	0.323	0.333	Proportion of true positives identified in the total sample (true positives/total cases).
Detection Prevalence	0.325	0.334	Proportion of positive predictions in the total sample (predicted positives/total cases).
Balanced Accuracy	0.82	0.829	Average of sensitivity and specificity, reflecting overall diagnostic balance.
False Positives (Incorrect Diagnoses)	3	3	Number of non-dyslipidemia cases incorrectly classified as dyslipidemia.
False Negatives (Missed Diagnoses)	348	330	Number of dyslipidemia cases incorrectly classified as no dyslipidemia.
McNemar’s Test *p*-Value	<0.001	<0.001	Indicates significant asymmetry in errors, primarily due to false negatives.
*p*-Value (Accuracy > NIR)	<0.001	<0.001	Significance of accuracy exceeding chance-level classification (NIR: 0.50).

## Data Availability

The data presented in this study are available on request from the corresponding author (J.W.) due to privacy and ethical concerns.

## Appendix A. Multifactor Model Specification

Level 1 (patient LDL measurements)

LDL_ij_ = β_0_ + β_1_(method_ij_) + β_2_(age_ij_) + β_3_(method_ij_ × age_ij_) + β_4_(sex_i_) + u_i_ + ϵ_ij_

where: i—patient id; j—LDL measurement for patient i; β_0_—is a constant, i.e., the average LDL level in the population; β_1_—coefficient for the method variable (main effect of LDL measurement method); β_2_—coefficient for the age variable (main effect of age); β_3_—coefficient for the interaction between method and age; β_4_—coefficient for the gender variable (main effect of gender); u_i_—random effect for patient i. which models the variability between patients; ϵ_ij_ are residuals (random errors) for measurement j within patient i.

Level 2 (for patients, random effect)

u_i_ ∼ *N*(0.τ^2^)

where τ^2^—variance of random effects between patients.

Random error (residuals for LDL measurements)

ϵ_ij_ ∼ *N*(0.σ^2^)

where σ^2^—variance of residuals for level 1 (within patient).
